# Association between dynapenic abdominal obesity and mild cognitive impairment among middle-aged and older community-dwelling adults

**DOI:** 10.1007/s40520-025-03135-z

**Published:** 2025-07-28

**Authors:** Lee Smith, Guillermo F. López Sánchez, Pinar Soysal, Nicola Veronese, Masoud Rahmati, Karel Kostev, Louis Jacob, Mark A. Tully, Fiona Richardson, Laurie Butler, Yvonne Barnett, Helen Keyes, Jae Il Shin, Ai Koyanagi

**Affiliations:** 1 Centre for Health Performance and Wellbeing, Anglia Ruskin University, Cambridge, UK; 2 Department of Public Health, Faculty of Medicine, Biruni University, Istanbul, Turkey; 3 Division of Preventive Medicine and Public Health, Department of Public Health Sciences, School of Medicine, University of Murcia, Murcia, Spain; 4 Department of Geriatric Medicine, Faculty of Medicine, Bezmialem Vakif University, Istanbul, Turkey; 5 Geriatric Unit, Department of Internal Medicine and Geriatrics, University of Palermo, Palermo, Italy; 6 Department of Physical Education and Sport Sciences, Faculty of Literature and Human Sciences, Lorestan University, Khoramabad, Iran; 7 Department of Physical Education and Sport Sciences, Faculty of Literature and Humanities, Vali-E-Asr University of Rafsanjan, Rafsanjan, Iran; 8 University Clinic of Marburg, Marburg, Germany; 9 Research and Development Unit, Parc Sanitari Sant Joan de Déu, Dr. Antoni Pujadas, Sant Boi de Llobregat, Barcelona, Spain; 10 AP-HP, Department of Physical Medicine and Rehabilitation, Université Paris Cité, Lariboisière-Fernand Widal Hospital, Paris, France; 11 U1153, Epidemiology of Ageing and Neurodegenerative Diseases (EpiAgeing), Inserm - Université Paris Cité, Paris, France; 12 School of Medicine, Ulster University, Londonderry, Northern Ireland UK; 13 The Queen Elizabeth Hospital King’s Lynn NHS Foundation Trust, King’s Lynn, PE30 4ET UK; 14 School of Psychology, Sport and Sensory Sciences, Anglia Ruskin University, Cambridge, UK; 15 Department of Pediatrics, Yonsei University College of Medicine, Seoul, South Korea; 16 Severance Underwood Meta-Research Center, Institute of Convergence Science, Yonsei University, Seoul, Republic of Korea

**Keywords:** Dynapenic abdominal obesity, Mild cognitive impairment, Older adults, Low- and middle-income countries

## Abstract

**Objectives:**

Dynapenic abdominal obesity (DAO) may potentially increase risk for mild cognitive impairment (MCI), but data is scarce, and community-based studies are lacking. Thus, we aimed to investigate the association between DAO and MCI in a large nationally representative community-based sample from six LMICs (China, Ghana, India, Mexico, Russia, South Africa).

**Methods:**

We analyzed cross-sectional data from the Study on Global Ageing and Adult Health. Dynapenia was defined as handgrip strength of < 26 kg for men and < 16 kg for women. Abdominal obesity was defined as waist circumference of > 88 cm for women and > 102 cm for men. DAO was defined as having both dynapenia and abdominal obesity. The National Institute on Ageing-Alzheimer’s Association criteria were used to define MCI. Multivariable logistic regression was performed.

**Results:**

Data on 32,715 individuals aged ≥ 50 years were analyzed [mean (SD) age 62.1 (15.6) years; 48.3% males]. Among those aged 50–64 years, dynapenia alone and DAO were significantly associated with MCI with the OR for DAO (OR = 1.79; 95%CI = 1.26–2.56) being higher than that of dynapenia alone (OR = 1.40; 95%CI = 1.15–1.71). In those aged ≥ 65 years, only dynapenia alone (OR = 1.53; 95%CI = 1.23–1.89) was significantly associated with MCI but not DAO. Abdominal obesity alone was not significantly associated with MCI in both age groups.

**Conclusions:**

Among community-dwellers in six LMICs, DAO was significantly associated with MCI among middle-aged individuals, but not among older people. However, it is important to note that the study was cross-sectional in nature, and thus, it is not known whether DAO leads to MCI or vice versa. Therefore, future longitudinal studies are necessary to clarify temporal associations and possible causality.

**Supplementary Information:**

The online version contains supplementary material available at 10.1007/s40520-025-03135-z.

## Introduction

Dementia is defined as a syndrome characterized by deterioration in cognitive function beyond what could be expected from normal ageing [1]. Globally, it is predicted that the prevalence of dementia will increase from 82 million in 2030 to 152 million in 2050 [1]. This increase is largely driven by rapid global ageing, since more people now are living into older age than ever before, while dementia increases exponentially with age [2]. Dementia is one of the principal causes of dependency and disability in older people, and has detrimental psychological, social, physiological, and economic impact on people living with the disease, their families, other care providers, and the entire society [3]. Unfortunately, there is currently no cure for dementia. Therefore, priority is placed on the identification of modifiable risk factors for prodromal stages of dementia to develop interventions aiming to prevent or delay the onset of dementia. For example, mild cognitive impairment (MCI) is a preclinical state of dementia with a high conversion rate to dementia (annual conversion rates ranging from 10 to 15% in clinical samples, and 3.8–6.3% in community-based samples) [4–6], and is increasingly being recognized as an important ‘target’ for the prevention of dementia.

To date, several risk factors for MCI have been identified [7], including low muscle strength and obesity [8–11]. However, there has been very little focus on the association of the co-existence of low muscle strength and obesity with cognitive impairment. It is possible for people with these two conditions to be at particularly high risk for cognitive decline owing to common pathological etiologies including inflammation, oxidative stress, and insulin resistance [12].

In particular, it is important to study the association between dynapenic abdominal obesity (DAO; i.e., impairment in muscle strength and high waist circumference) [13] and MCI as DAO is now considered an important risk concept associated with higher risk for metabolic syndrome, cardiovascular diseases, type II diabetes [14], disability, and premature mortality [15]. However, to the best of the authors’ knowledge, there is currently only one study on the association between DAO and MCI. Specifically, in one small cross-sectional study including 417 older outpatients from Japan with cardiometabolic diseases, it was found that patients with DAO were at increased odds for MCI (odds ratio [OR] = 3.98; 95% confidence interval [CI] = 1.15–13.77). Moreover, low handgrip strength (OR = 2.19; 95%CI = 1.11–4.29) and high waist circumference (OR = 2.0; 95%CI = 1.03–3.99) alone were also associated with MCI [12]. Clearly, more research is needed in larger samples of community-dwelling older adults from other global regions to either confirm or refute findings from the single study on this important topic. In particular, low- and middle-income countries (LMICs) are apposite settings to investigate this association as the speed of ageing in LMICs is outpacing that of high-income countries, and it is possible for conditions such as DAO and dementia, which are strongly related with ageing, to cause a substantial burden in this setting. Indeed, by 2050, the United Nation projects that two-thirds of the world’s population aged 60 years and over will be living in LMICs [2].

Given this background, the aim of the present study was to investigate the cross-sectional association between DAO and MCI in 32,715 community-dwelling adults aged ≥ 50 years from six LMICs. It is important to note that due to the cross-sectional nature of this study, the aim is not to establish causality.

## Methods

### The survey

Data from the Study on Global Ageing and Adult Health (SAGE) were analyzed. This survey was conducted in China, Ghana, India, Mexico, Russia, and South Africa between 2007 and 2010. Based on the World Bank classification at the time of the survey, all these countries were LMICs. Details of the survey methodology have been published elsewhere [16]. Briefly, to obtain nationally representative samples, a multistage clustered sampling design method was applied. The sample consisted of adults aged ≥ 18 years with oversampling of those aged ≥ 50 years. Trained interviewers conducted face-to-face interviews using a standard questionnaire. Standard translation procedures were undertaken to guarantee comparability between countries. The survey response rates were: China 93%; Ghana 81%; India 68%; Mexico 53%; Russia 83%; and South Africa 75%. Sampling weights were constructed to adjust for the population structure as reported by the United Nations Statistical Division. Ethical approval was obtained from the WHO Ethical Review Committee and local ethics research review boards. Written informed consent was obtained from all participants.

### Mild cognitive impairment

MCI was ascertained based on the recommendations of the National Institute on Aging-Alzheimer’s Association [17]. We applied the identical algorithms used in previous SAGE publications using with the same survey questions to identify MCI [18, 19]. Briefly, individuals fulfilling all of the following conditions were considered to have MCI:

- *Concern about a change in cognition*: Individuals who replied ‘bad’ or ‘very bad’ to the question “How would you best describe your memory at present?” and/or those who answered ‘worse’ to the question “Compared to 12 months ago, would you say your memory is now better, the same or worse than it was then?” were considered to have this condition.
- *Objective evidence of impairment in one or more cognitive domains*: was based on a <-1 SD cut-off after adjustment for level of education, age, and country. Cognitive function was assessed through the following performance tests: word list immediate and delayed verbal recall from the Consortium to Establish a Registry for Alzheimer’s Disease [20], which assessed learning and episodic memory; digit span forward and backwards from the Weschler Adult Intelligence Scale [21], that evaluated attention and working memory; and the animal naming task [22], which assessed verbal fluency.
- *Preservation of independence in functional abilities*: was assessed by questions on self-reported difficulties with basic activities of daily living (ADL) in the past 30 days [23]. Specific questions were: “How much difficulty did you have in getting dressed?” and “How much difficulty did you have with eating (including cutting up your food)?” The answer options were none, mild, moderate, severe, and extreme (cannot do). Those who answered either none, mild, or moderate to both of these questions were considered to have preservation of independence in functional activities. All other individuals were deleted from the analysis (935 individuals aged ≥ 50 years).
- *No dementia*: Individuals with a level of cognitive impairment severe enough to preclude the possibility to undertake the survey were not included in the current study.

### Dynapenia, abdominal obesity and dynapenic abdominal obesity

Handgrip strength was measured using a Smedley Hand Dynamometer (Scandidact Aps, Denmark). Dynapenia was defined as < 26 kg for men and < 16 kg for women [24], using the average value of the two handgrip measurements of the dominant hand. Waist circumference was measured at the midpoint between the lower margin of the lowest palpable rib and the top of the iliac crest keeping the measuring tape parallel to the floor. Abdominal obesity was defined as a waist circumference of > 88 cm for women and > 102 for men [25]. Participants were divided into four groups according to dynapenia and abdominal obesity status: No dynapenia and no abdominal obesity, dynapenia alone, abdominal obesity alone, and DAO (i.e., dynapenic abdominal obesity).

### Control variables

The selection of the control variables was based on past literature [8, 9], and included age, sex, country-wise wealth quintiles based on income, education (years), smoking (never, current, past), physical activity, alcohol consumption, diabetes, stroke, and hypertension.

Levels of physical activity were assessed with the Global Physical Activity Questionnaire and were classified as low, moderate, and high based on conventional cut-offs [26]. Consumers of at least four (females) or five drinks (males) of any alcoholic beverage per day on at least one day in the past week were considered ‘heavy’ drinkers. Those who had ever consumed alcohol but were not heavy drinkers were categorized as ‘non-heavy drinkers’ [27]. Diabetes and stroke were solely based on lifetime self-reported diagnosis. Hypertension was defined as having at least one of the following: systolic blood pressure ≥ 140 mmHg; diastolic blood pressure ≥ 90 mmHg; or self-reported diagnosis.

### Statistical analysis

The statistical analysis was performed with Stata 14.2 (Stata Corp LP, College station, Texas). The analysis was restricted to those aged ≥ 50 years as MCI is an age-related condition. The difference in sample characteristics among those with and without MCI were tested by Chi-squared tests for categorical variables and Student’s *t*-tests for continuous variables. Multivariable logistic regression analysis was performed to examine the association between the four-category variable on dynapenia, abdominal obesity, or both (exposure) and MCI (outcome), with no dynapenia and no abdominal obesity being the reference category. Age-stratified analyses (i.e., 50–64, ≥ 65 years) were also conducted as previous studies have shown that the correlates of MCI may differ between middle-aged and older people, especially in terms of body mass [7, 9]. All regression analyses were adjusted for age, sex, wealth, education, smoking, physical activity, alcohol consumption, diabetes, stroke, hypertension, and country. Adjustment for country was done by including dummy variables for each country in the model as in previous SAGE publications [18, 28]. The sample weighting and the complex study design were taken into account in the analyses. Results from the regression analyses are presented as odds ratios (ORs) with 95% confidence intervals (CIs). The level of statistical significance was set at *P* < 0.05.

## Results

**Fig. 1:**
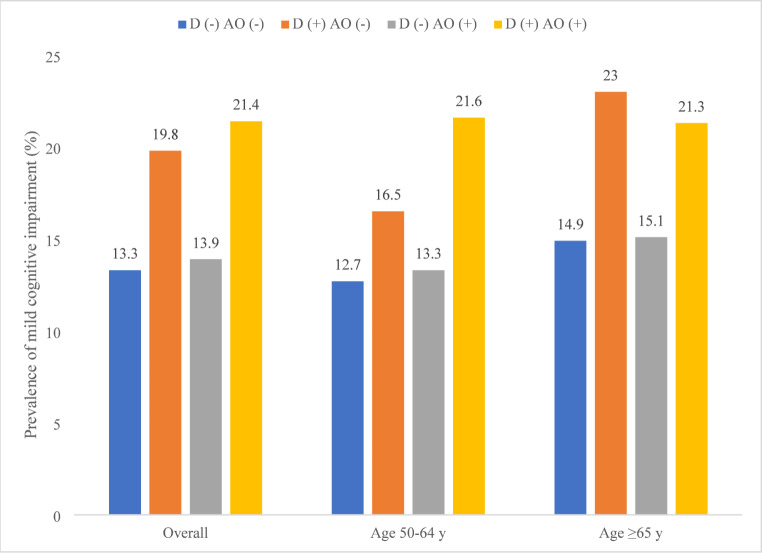
Prevalence of mild cognitive impairment by dynapenia and abdominal obesity status (overall and by age groups) Abbreviation: D Dynapenia; AO Abdominal obesity

Data on 32,715 individuals aged ≥ 50 years with preservation of independence in functional abilities were analyzed (China *n* = 12,815; Ghana *n* = 4,201; India *n* = 6,191; Mexico *n* = 2,070; Russia *n* = 3,766; South Africa *n* = 3,672). The prevalence of MCI was 15.3%, while that of dynapenia alone, abdominal obesity alone, and DAO were 25.2%, 16.9%, and 5.7%, respectively. The sample characteristics by MCI status are provided in Table 1. The mean (SD) age was 62.1 (15.6) years and 48.3% were males. Those with MCI were significantly older, more likely to be females, and had lower levels of education and wealth. Furthermore, they were more likely to have low levels of physical activity, and have stroke and hypertension. The sample characteristics by DAO status are shown in Table S1 of the Appendix. Those with DAO were more likely to be older and have more physical diseases such as diabetes, stroke, and hypertension. The prevalence of MCI by dynapenia and abdominal obesity status are shown in Fig. 1 (details on 95%CIs are provided in Table S2 of the Appendix). The prevalence of MCI was particularly high in those with dynapenia alone (19.8%) or DAO (21.4%) in the overall sample (Fig. 1). The difference in the prevalence of MCI between dynapenia alone (16.5%) and DAO (21.6%) was more pronounced among those aged 50–64 years than in those aged ≥ 65 years, in which the prevalence of MCI among these two conditions were similar. After adjustment for potential confounders, in the overall sample, dynapenia alone (OR = 1.48; 95%CI = 1.27–1.73) and DAO (OR = 1.45; 95%CI = 1.11–1.88) were both significantly associated with higher odds for MCI, but abdominal obesity alone was not (Table 2). Among those aged 50–64 years, similar to the overall sample, only dynapenia alone and DAO were significantly associated with MCI but the OR for DAO (OR = 1.79; 95%CI = 1.26–2.56) was higher than that of dynapenia alone (OR = 1.40; 95%CI = 1.15–1.71). Finally, among those aged ≥ 65 years, only dynapenia alone (OR = 1.53; 95%CI = 1.23–1.89) was significantly associated with MCI but not abdominal obesity alone or DAO.

**Table 1:** Sample characteristics (overall and by mild cognitive impairment status)

			Mild cognitive impairment		
Characteristic		Overall	No (*n* = 26771)	Yes (*n* = 4854)	P-value^a^
Age (years)	Mean (SD)	62.1 (15.6)	61.7 (15.2)	64.4 (17.2)	< 0.001
Sex	Female	51.7	51.4	55.1	0.006
	Male	48.3	48.6	44.9	
Education (years)	Mean (SD)	6.1 (8.9)	6.2 (9.1)	4.8 (7.5)	< 0.001
Wealth	Poorest	16.9	16.1	22.6	< 0.001
	Poorer	18.9	18.5	22.8	
	Middle	19.4	18.6	24.5	
	Richer	21.5	22.0	18.6	
	Richest	23.3	24.9	11.5	
Smoking	Never	58.7	58.2	59.0	0.381
	Current	34.9	35.5	34.1	
	Past	6.4	6.4	6.9	
Physical activity	High	49.9	51.1	47.7	< 0.001
	Moderate	23.0	23.4	19.6	
	Low	27.1	25.5	32.7	
Alcohol consumption	Never	66.7	66.3	67.1	0.006
	Non-heavy	29.1	29.7	27.2	
	Heavy	4.2	4.0	5.7	
Diabetes	No	93.3	93.4	93.1	0.703
	Yes	6.7	6.6	6.9	
Stroke	No	97.3	97.7	95.1	< 0.001
	Yes	2.7	2.3	4.9	
Hypertension	No	45.2	46.1	39.0	< 0.001
	Yes	54.8	53.9	61.0	

Abbreviation: SD Standard deviation

Data are % unless otherwise stated

^a^ P-value was obtained by Chi-squared tests for categorical variables and Student’s *t*-tests for continuous variables

**Table 2:** Association between dynapenia, abdominal obesity, or both, and mild cognitive impairment (outcome) estimated by multivariable logistic regression

	Overall		Age 50–64 years		Age ≥ 65 years	
Exposure	OR	95%CI	OR	95%CI	OR	95%CI
D (-) AO (-)	1.00		1.00		1.00	
D (+) AO (-)	1.48***	[1.27,1.73]	1.40**	[1.15,1.71]	1.53***	[1.23,1.89]
D (-) AO (+)	1.12	[0.93,1.35]	1.21	[0.97,1.52]	0.98	[0.70,1.36]
D (+) AO (+)	1.45**	[1.11,1.88]	1.79**	[1.26,2.56]	1.29	[0.92,1.80]

Abbreviation: OR Odds ratio; CI Confidence interval; D Dynapenia; AO Abdomial obesity

Model are adjusted for age, sex, wealth, education, smoking, physical activity, alcohol consumption, diabetes, stroke, hypertension, and country

* *p* < 0.05, ** *p* < 0.01, *** *p* < 0.001

## Discussion

In our study including large representative samples of middle-aged (defined in this study as those aged 50–64 years) and older adults (defined in this study as those aged ≥ 65 years) from LMICs, we found that the association between DAO and MCI differs by age. Specifically, in the adjusted model, compared to no dynapenia and no abdominal obesity, DAO was significantly associated with 1.79 (95%CI = 1.26–2.56) times higher odds for MCI among adults aged 50–64 years but among adults aged ≥ 65 years, this association was not statistically significant (OR = 1.29; 95%CI = 0.92–1.80). Interestingly, in both age groups, dynapenia alone was significantly associated with 1.40–1.53 times higher odds for MCI but abdominal obesity alone was not significantly associated with MCI. To the best of our knowledge, this is the first study on DAO and MCI in the general population.

The findings of our study are partially in line with the only previous study on DAO and MCI conducted in a small sample of older adults with cardiometabolic disease from Japan [12]. Specifically, in this Japanese study, dynapenia alone, abdominal obesity alone, and DAO were all significantly associated with MCI, while in our study, abdominal obesity alone was not significantly associated with MCI. Although the reasons for this discrepancy are unclear, it is possible that this is related to the fact that the Japanese study only included people with cardiometabolic diseases.

There are several plausible mechanisms that may explain the DAO-MCI association. First, as previously mentioned, this relationship may be mediated by insulin resistance and inflammation. Indeed, DAO has been found to be associated with an unfavorable inflammatory profile [29], and inflammation has been implicated in the development of MCI, specifically C-reactive proteins and Interleukin-6 [30]. Moreover, low-grade inflammation promotes insulin resistance [31], and this may lead to cognitive decline via impaired insulin signaling [32]. Second, DAO has been found to lead to worsening of activities of daily living (ADL) disability prospectively [33], and it is possible for people with ADL disabilities to have difficulties to participate in cognitively stimulating activities, and this may lead to cognitive decline.

Interestingly, in our study, although DAO was associated with higher odds for MCI among middle-aged people, DAO was not significantly associated with MCI among older individuals. This may be explained by the difference in the effect of being overweight/obese on cognitive function by age that has been previously reported. Indeed, some studies have found that being overweight/obese may be a risk factor for cognitive decline or dementia in middle age, but that being overweight/obese could even be a protective factor in older age [34]. While the reasons for this are unclear, it could be that being overweight in older adulthood (following periods of relatively normal weight throughout life) may impact cognition less than being overweight starting earlier in the life course [35]. For example, in a review of the literature focusing on the relationship between obesity and aging and how these interact together to affect cognitive function, results suggested that effects are likely mediated by the accelerated effects obesity has on the integrity of neural structures, including both gray and white matter. Moreover, epidemiological studies have provided evidence that obesity in mid-life is linked to an increased risk for Alzheimer’s Disease and vascular dementia, most likely via an increased accumulation of Alzheimer’s Disease pathology [34]. Alternatively, older adults who have a higher body fat mass are often less likely to have frailty or other comorbidities, and this may have a protective effect on cognitive impairment [36]. This may explain why the effect of DAO on MCI was attenuated in older people in our study.

However, there is a scarcity of longitudinal studies on this topic, and thus, future studies of this nature are required to elucidate on the DAO-MCI association by age.

The analysis of large representative samples of middle-aged and older adults from six LMICs is a clear strength of the present study. However, findings must be interpreted in light of the study limitations. First, the study was cross-sectional in nature, and thus, it is not known whether DAO leads to MCI or vice versa. For example, it is possible that those with MCI are less likely to engage in social activities, and also consume an unfavorable diet, that consequently results in DAO. Thus, future longitudinal studies are necessary to understand the direction of the association. Second, the present study utilized dynapenia cut-points suggested by Studenski et al. [24], and these cut-points were derived based on large diverse populations. However, the present paper utilized a large multiethnic sample from LMICs, and thus, a different population to Studenski et al. It is therefore possible that the cut-points used in this study may have over- or underestimated dynapenia. Next, individuals at early stages of dementia could have been included in the MCI group based on our definition of independence in functional abilities. However, since there is no consensus in terms of the acceptable level of functional impairment that MCI individuals could manifest, we deemed that it would be better to maintain a more conservative definition of preservation of basic independence in line with previous studies [37]. Finally, given that the institutionalized were excluded from the survey, our study results cannot be generalized to this population that could have higher prevalence of DAO and MCI.

In conclusion, in the present study using large representative samples of adults aged ≥ 50 years from six LMICs, DAO was associated with significantly increased odds for MCI only among the middle-aged. Future studies from other regions are necessary to assess whether our findings can be replicated especially in terms of the age differences. Furthermore, longitudinal studies on this topic may shed light on temporal associations and possible causality.

## Electronic supplementary material

Below is the link to the electronic supplementary material.

Supplementary Material 1

## Data Availability

No datasets were generated or analysed during the current study.
