# Supplementary material for: Association between dynapenic abdominal obesity and mild cognitive impairment among middle-aged and older community-dwelling adults
Source: Aging Clin Exp Res. 2025 Jul 28;37(1):235. doi: 10.1007/s40520-025-03135-z (PMC12304052; doi:10.1007/s40520-025-03135-z)
Supplement: Supplementary file 1 — Supplementary Material 1 [file 40520_2025_3135_MOESM1_ESM.docx]

| **Table S1** Sample characteristics by dynapenia and abdominal obesity status | | | | | | |
| --- | --- | --- | --- | --- | --- | --- |
|  |  | Dynapenia and adbominal obesity status | | | |  |
| Characteristics |  | D(-)AO(-) | D(+)AO(-) | D(-)AO(+) | D(+)AO(+) | P-value |
| Age (years) | Mean (SD) | 60.1 (14.6) | 65.0 (15.7) | 61.3 (16.9) | 65.5 (17.3) | <0.001 |
| Sex | Female | 40,3 | 41,6 | 85,6 | 86,4 | <0.001 |
|  | Male | 59,7 | 58,4 | 14,4 | 13,6 |  |
| Education (years) | Mean (SD) | 6.0 (8.9) | 4.4 (7.8) | 7.0 (10.3) | 5.5 (9.3) | <0.001 |
| Wealth | Poorest | 16,7 | 22,0 | 11,1 | 13,0 | <0.001 |
|  | Poorer | 18,3 | 21,3 | 15,8 | 17,0 |  |
|  | Middle | 19,1 | 20,2 | 20,0 | 18,9 |  |
|  | Richer | 22,6 | 18,3 | 24,5 | 23,0 |  |
|  | Richest | 23,3 | 18,1 | 28,7 | 28,2 |  |
| Smoking | Never | 50,4 | 50,2 | 81,4 | 78,5 | <0.001 |
|  | Current | 42,7 | 43,2 | 13,9 | 17,2 |  |
|  | Past | 6,9 | 6,6 | 4,7 | 4,3 |  |
| Physical activity | High | 57,6 | 41,4 | 53,1 | 33,1 | <0.001 |
|  | Moderate | 21,8 | 26,3 | 21,7 | 28,6 |  |
|  | Low | 20,6 | 32,2 | 25,2 | 38,3 |  |
| Alcohol consumption | Never | 63,2 | 74,4 | 68,4 | 83,2 | <0.001 |
|  | Non-heavy | 31,0 | 22,7 | 29,8 | 15,8 |  |
|  | Heavy | 5,8 | 2,9 | 1,9 | 1,0 |  |
| Diabetes | No | 94,7 | 95,1 | 89,1 | 86,4 | <0.001 |
|  | Yes | 5,3 | 4,9 | 10,9 | 13,6 |  |
| Stroke | No | 98,2 | 96,4 | 97,5 | 95,5 | <0.001 |
|  | Yes | 1,8 | 3,6 | 2,5 | 4,5 |  |
| Hypertension | No | 50,6 | 53,9 | 27,1 | 23,3 | <0.001 |
|  | Yes | 49,4 | 46,1 | 72,9 | 76,7 |  |
| Abbreviation: D Dynapenia; AO Abdominal obeisty; SD Standard deviation Data are % unless otherwise stated.  P-value was obtained by Chi-squared tests for categorical variables and one-way ANOVA for continuous variables. | | | | | | |

| **Table S2** Prevalence of mild cognitive impairment by dynapenia and abdominal obesity status with 95% confidence intervals (overall and by age groups) | | |
| --- | --- | --- |
| Overall | % | 95%CI |
| D (-) AO (-) | 13,3 | [12.2,14.6] |
| D (+) AO (-) | 19,8 | [17.5,22.2] |
| D (-) AO (+) | 13,9 | [12.0,16.0] |
| D (+) AO (+) | 21,4 | [17.4,26.2] |
| Age 50-64 years |  |  |
| D (-) AO (-) | 12,7 | [11.5,14.0] |
| D (+) AO (-) | 16,5 | [13.2,20.3] |
| D (-) AO (+) | 13,3 | [11.1,15.8] |
| D (+) AO (+) | 21,6 | [15.6,29.1] |
| Age ≥65 years |  |  |
| D (-) AO (-) | 14,9 | [13.3,16.7] |
| D (+) AO (-) | 23.0 | [20.4,25.9] |
| D (-) AO (+) | 15,1 | [12.1,18.6] |
| D (+) AO (+) | 21,3 | [17.1,26.2] |
| Abbreviation: Dynapenia; AO Abdominal obesity; CI Confidence interval | | |
